# Brazilian propolis ethanol extract and its component kaempferol induce myeloid-derived suppressor cells from macrophages of mice *in vivo* and *in vitro*

**DOI:** 10.1186/s12906-018-2198-5

**Published:** 2018-05-02

**Authors:** Hiroshi Kitamura, Natsuko Saito, Junpei Fujimoto, Ken-ichi Nakashima, Daisuke Fujikura

**Affiliations:** 1 Laboratory of Veterinary Physiology, Department of Veterinary Medicine, School of Veterinary Medicine, Rakuno Gakuen University, 582 Bunkyodai-Midorimachi, Ebetsu, Hokkaido 069-8501 Japan; 2 Laboratory of Medicinal Resources, School of Pharmacy, Aichi Gakuin University, 1-100 Kusumoto-cho, Chikusa-ku, Nagoya, Aichi, 464-8650 Japan; 3 Center for Advanced Research and Education, Asahikawa Medical University, 2-1-1-1 Midorigaoka-Higashi, Asahikawa, Hokkaido 078-8510 Japan

**Keywords:** Propolis, Kaempferol, Myeloid-derived suppressor cells, Macrophage, Type 2 diabetes, Chronic inflammation

## Abstract

**Background:**

Brazilian green propolis is produced by mixing secretions from Africanized honey bees with exudate, mainly from *Baccharis dracunculifolia.* Brazilian propolis is especially rich in flavonoids and cinammic acid derivatives, and it has been widely used in folk medicine owing to its anti-inflammatory, anti-viral, tumoricidal, and analgesic effects. Moreover, it is applied to prevent metabolic disorders, such as type 2 diabetes and arteriosclerosis. Previously, we demonstrated that propolis ethanol extract ameliorated type 2 diabetes in a mouse model through the resolution of adipose tissue inflammation. The aims of this study were to identify the immunosuppressive cells directly elicited by propolis extract and to evaluate the flavonoids that induce such cells.

**Methods:**

Ethanol extract of Brazilian propolis (PEE; 100 mg/kg i.p., twice a week) was injected into lean or high fat-fed obese C57BL/6 mice or C57BL/6 *ob/ob* mice for one month. Subsequently, immune cells in visceral adipose tissue and the peritoneal cavity were monitored using FACS analysis. Isolated macrophages and the macrophage-like cell line J774.1 were treated with PEE and its constituent components, and the expression of immune suppressive myeloid markers were evaluated. Finally, we injected one of the identified compounds, kaempferol, into C57BL/6 mice and performed FACS analysis on the adipose tissue.

**Results:**

Intraperitoneal treatment of PEE induces CD11b^+^, Gr-1^+^ myeloid-derived suppressor cells (MDSCs) in visceral adipose tissue and the peritoneal cavity of lean and obese mice. PEE directly stimulates cultured M1 macrophages to transdifferentiate into MDSCs. Among twelve compounds isolated from PEE, kaempferol has an exclusive effect on MDSCs induction *in vitro*. Accordingly, intraperitoneal injection of kaempferol causes accumulation of MDSCs in the visceral adipose tissue of mice.

**Conclusion:**

Brazilian PEE and its compound kaempferol strongly induce MDSCs in visceral adipose tissue at a relatively early phase of inflammation. Given the strong anti-inflammatory action of MDSCs, the induction of MDSCs by PEE and kaempferol is expected to be useful for anti-diabetic and anti-inflammatory therapies.

**Graphical Abstract:**

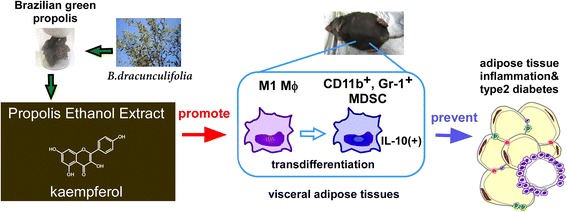

**Electronic supplementary material:**

The online version of this article (10.1186/s12906-018-2198-5) contains supplementary material, which is available to authorized users.

## Background

Low-grade inflammation in adipose tissue is a hallmark of type 2 diabetes and adiposity [1]. Adipose tissue inflammation is attributed to aberrant proportions of resident macrophages: an increase in inflammation-promoting M1 macrophages and a decrease in anti-inflammatory M2 macrophages [2, 3]. The imbalance of adipose tissue macrophages is triggered by cytokines released by enlarged adipocytes, as well as an accumulation of immune cells [4]. On the other hand, type 2 innate lymphoid cells and their target eosinophils repress M1 macrophage activation in adipose tissue, and subsequently attenuate metabolic disorders [5, 6]. Moreover, regulatory T cells inhibit chronic inflammation by the secretion of anti-inflammatory cytokines, such as interleukin (IL)-10 and transforming growth factor (TGF)-β1 [7, 8]. Myeloid derived-suppressor cells (MDSCs) are another cell type that has the potential to modulate inflammation [9–11]. MDSCs, as their name represents, are known to be derived from myeloid lineage progenitors, and intensely express both CD11b and Gr-1 as cell surface markers in mouse [9, 10]. MDSCs produce vast amounts of IL-10 and arginase, resulting in strong attenuation of immune activation in tumors and septic regions [9, 10]. Previously, CD11b^+^, Gr-1^+^ MDSCs were shown to inhibit the progression of type 2 diabetes in obese mice [12]. Although manipulation of MDSCs is beneficial in anti-diabetic treatment, an induction procedure for MDSCs has not yet been established.

Propolis is a resinous mixture that honey bees produce by mixing their waxes and saliva with exudate from botanical substances. Thus, the composition of propolis is determined by geographic location and the bee’s genetics [13, 14]. Brazilian green propolis is produced by Africanized bees, which are hybrids between *Apis mellifera scutellata* (African honey bee) and *Apis mellifera* (European honey bee), and is rich in organic substances mainly from *Baccharis dracunculifolia* [15, 16]*.* Brazilian propolis ethanol extract (PEE) exerts various biological effects, including tumoricidal, immunomodulatory, and tissue repair effects [17–20]. Some of these effects are considered to be attributable to cinnamic acid derivatives and flavonoids, including artepillin C, p-coumaric acid, caffeic acid phenethyl ester (CAPE), and kaempferol [21–24]. Traditionally, supplements containing propolis are taken to prevent the progression of metabolic disorders. In agreement with this type of propolis use, orally administration of propolis was evinced to have potential to restore type 2 diabetes in human [25, 26]. Previously, we found that intraperitoneal injection of PEE ameliorates type 2 diabetes in *ob/ob* mice, although it did not influence the body weight gain of the mice [27]. In these mice, PEE modulated the number of eosinophils and M1 macrophages in mesenteric adipose tissue where adiposity is closely associate with insulin sensitivity. However, it is still elusive which cells are direct targets of propolis in the adipose tissue. In this study, we found that PEE induces MDSCs in adipose tissue. We attempted to identify cells which are source of PEE-elicited MDSCs. We also tried to identify chemicals which increase MDSCs *in vitro* and *in vivo.*

## Methods

### PEE

Brazilian PEE was provided by the Yamada Bee Farm (Kagamino, Japan). Preparation of PEE was conducted as follows: Brazilian green propolis was homogenized in a 10-fold volume of ethanol and agitated at room temperature for 12 h. Subsequently the solution was filtrated and evaporated until the solid content of the solution was 55% by weight. Total solid content was determined after evaporation in vacuo. The chemical composition of this lot (lot no. 550703) is listed in Table 1.

**Table 1:** Chemical composition of Brazilian green propolis ethanol extract used in this study (Lot. 55,703)

Chemicals	wt%
artepillin C	9.5
baccharin	3.5
kaempferide	1.7
drupanin	1.4
p-coumaric acid	1.2
culifolin	0.23
caffeic acid phenethyl ester	0.14
chlorogenic acid	0.12
kaempferol	0.11
pinocembrin	0.037
narigenin	0.019
chrysin	0.0029

### Animals and treatment

Male C57BL/6JHamSlc-*ob/ob* mice (*ob/ob* mice) and C57BL/6NCrSlc mice (C57BL/6 mice) were purchased from Japan SLC (Hamamatsu, Japan). The mice were housed in positive ventilated cages (Allentown, Allentown, NJ, USA) under conventional conditions at 24 ± 1 °C on a 12-h light-dark cycle (lights on from 8:00–20:00) and were given free access to food and water. After purchase, the mice were acclimatized for at least five days before the experimental period. C57BL/6 mice were fed a 60% kcal high-fat diet (HFD; D12492; Research diets, New Brunswick, NJ, USA) or a normal diet (Labo MR Stock; Nosan Corporation, Yokohama, Japan) from the age of five weeks. During the experimental period, the mice were kept in separate cages to avoid fighting. There were two treatment groups with intraperitoneal injections in both lean and obese mice. The first group involved treatment with PEE (100 mg/kg i.p., twice weekly) or HPLC-qualified kaempferol (PubChem CID5280863; > 95% purity quantified by HPLC; Wako, Osaka, Japan; cat. no. 110–00451; 1 or 10 mg/kg i.p., twice weekly). The second group involved treatment with a vehicle (PBS containing ethanol and DMSO, twice weekly) using a 1 mL syringe attached to a 23-gauge needle. Mice were treated with PEE or kaempferl for one (PEE) or two (kaempferol) months from six weeks of age (lean and *ob/ob* mice) or 17 weeks of age (HFD-fed obese mice weighing more than 40 g). The reagents were administrated to mice in the Treatment Room of the Laboratory Animal Station of Rakuno Gakuen University between 11:00 and 13:00. We randomly allocated mice to the treatment groups at the beginning of the experiment. No statistical differences in body weight were observed between the different treatment groups. The average weights of the lean and HFD-fed obese C57BL/6 mice and the *ob/ob* mice at the beginning and end of the treatment regimens are listed in Additional file 1. Blood glucose levels of the mice are listed in Additional file 2. The mice in the different groups were treated alternately. All mice were eventually anesthetized and sacrificed by cervical dislocation, and the peritoneal fluid, bone marrow, and mesenteric and epididymal adipose tissue were collected for quantitative fluorescence-activated cell sorting (FACS) and quantitative reverse transcription polymerase chain reaction (qRT-PCR) analyses. In a previous study, 4–7 mice were required in each experimental group to investigate the effects of PEE on adipose tissue immune cells [27]. In this study, we used four mice in each treatment group for quantitative analysis. The Ethics Review Committee for Animal Experimentation periodically performed animal welfare checks during assessments and interventions. Veterinarians or veterinary students checked the health status of all animals once daily. The ARRIVE Guidelines checklist of this study can be downloaded.

### Blood biochemistry tests

Blood glucose levels were determined using a FreeStyle Freedom Lite meter (Abott Laboratories, Abott Park, IL, USA). Blood triglycerides, total cholesterol, and non-esterified fatty acids were measured using Test Wako kits (Wako, Osaka, Japan).

### FACS analysis

Preparation of the vascular stromal fraction (VSF) of adipose tissue has been described previously [27]. Fc receptors on the cells in VSF and peritoneal cells were blocked by TruStain FcX (BioLegend, San Diego, CA, USA), and then labeled with phycoerythrin-conjugated F4/80 (BioLegend), AlexaFluor488-conjugated CD11b (Biolegend), allophycocyanin-conjugated Gr-1 (BioLegend) or phycoerythrin-conjugated Siglec-F (BD Bioscience). After washing with PBS, dead cells were stained with 7-amino-actinomycin D (BioLegend), and monitored using FACS Canto II (BD Bioscience), FACSVerse (BD Bioscience) or MoFlo Astrios (Beckman Coulter, Indianapolis, IN, USA). FACS data were subsequently analyzed using FlowJo software (Tree Star). After gating with plot patterns of forward scatter and side scatter, eosinophils (CD11b^mid,^ Siglec-F^+^), macrophages (F4/80^+^, CD11b^+^, Gr-1^−^), and MDSCs (F4/80^+^, CD11b^+^, Gr-1^+^) were identified and quantified. The numerical data and gating processes of the FACS analysis are shown in Additional files 3 and 4, respectively.

### Cells and treatment

Peritoneal macrophages were prepared from thioglycolate medium-elicited (2 mL/head i.p.; Sigma Aldrich, St Louis, MI, USA) or intact mice as described previously [28]. Mouse peritoneal macrophages and mouse macrophage-like cell line J774.1 (RIKEN BioResource Center, Tsukuba, Japan) were seeded at a density of 5.0 × 10^5^/mL and cultured in RPMI1640 medium containing 10% fatal calf serum (FCS), penicillin (100 U/mL, Nacalai), and streptomycin (100 μg/mL, Nacalai). Bone marrow macrophages were isolated as previously described [29]. Briefly, femur bone marrow was isolated and monocytes were collected using 14.4% NycoPrep (Axis-shield, Dundee, Scotland). Monocytes were cultured in Dulbecco’s modified Eagle medium (DMEM) containing 4.5 g/L of glucose, 10% FCS, penicillin (100 U/mL, Nacalai), and streptomycin (100 μg/mL, Nacalai). After culturing for 24 h, suspended cells were differentiated in DMEM containing granulocyte macrophage-colony stimulating factor (GM-CSF, 50 μg/mL; PeproTech, Rocky Hill, USA) for M1 macrophages or macrophage-colony stimulating factor (M-CSF, 50 μg/mL; PeproTech) for M2 macrophages for seven days.

PEE, baccharin (PubChem CID 9947201, > 98% purity qualified by HPLC, Yamada Bee Farm, lot no. TB081104S), culifolin (PubChem CID 9861070, > 98% purity qualified by HPLC, Yamada Bee Farm, lot no. YKH247B-1), p-coumaric acid (PubChem CID 637542, Sigma Aldrich, > 98% purity quantified by HLPC, lot no. 078 K1386), kaempferol (> 95% purity quantified by HPLC, Wako, lot no. ACK5342), chlorogenic acid (PubChem CID 1794427, > 95% purity quantified by HPLC, Sigma Aldrich, lot no. 096 K1722), chrysin (PubChem CID 5281607, > 98% purity quantified by HPLC, Wako, lot no. YKH247B-1), naringenin (PubChem CID 439246, > 95% purity quantified by HPLC, Sigma Aldrich, lot no. 056 K1362), and pinocembrin (PubChem CID 238782, > 95% purity quantified by HPLC, Sigma Aldrich, lot no. 075 K1076) were kindly gifted by Yamada Bee Farm. Chemical synthesis procedures of baccharin and culifolin were described previously [30]. Drupanin (PubChem CID 6440361) was prepared as previously described [31]. Artepillin C (PubChem CID 5472440, > 98% purity qualified by HPLC, cat. no. 019–26,711) and CAPE (PubChem CID 5281787, > 97% purity qualified by HPLC, cat. no. C8221) were purchased from Wako and Sigma Aldrich, respectively. All chemicals were diluted in DMSO and RPMI1640 medium, and macrophages were treated for 24 h. The concentrations of compounds were: PEE, 1, 10, or 100 μg/mL; artepillin C, 9.5 μg/mL; baccharin, 3.5 μg/mL; CAPE, 0.14 μg/mL; chlorogenic acid, 0.12 μg/mL; chrysin, 2.9 ng/mL; culifolin, 0.23 μg/mL; drupanin, 1.4 μg/mL; kaempferol, 0.03, 0.1, or 1.0 μg/mL; kaempheride, 1.7 μg/mL; naringenin, 0.019 μg/mL; p-coumaric acid, 1.2 μg/mL; and pinocembrin, 0.037 μg/mL.

### qRT-PCR analysis

Total RNA extracted using RNAiso Plus (Takara bio, Otsu, Japan) was subjected to qRT-PCR analysis as described previously [32]. A quantitative PCR reaction was performed with KAPA SYBR FAST qPCR Master Mix (KAPA Biosystems, Wilmington, MA, USA) or the SensiFAST SYBR No-ROX kit (Bioline, Taunton, MA, USA) using an ECO qPCR system (Illumina, San Diego, CA, USA). The following primers were used: 5’-ATTGTATTGGGGTCCCACCT-3′ and 5’-GTCCAGAGTAGTGGGGCAGA-3′ for *Ly6g*; 5’-TGCTATGCTGCCTGCTCTTA-3′ and 5’-TCATTTCCGATAAGGCTTGG-3′ for *Il10;* and 5’-TCATTATGCCGAGGATTTGG-3′ and 5’-ACTTTTATGTCCCCCGTTGA-3′ for *Hprt-1*. TaqMan mixture for 18 s rRNA were purchased from Thermo Fisher Scientific (Waltham, MA, USA). The raw qRT-PCR data are shown in Additional file 3.

### Statistical analysis

Statistical analysis was performed using a Student’s *t-*test or one-way analysis of variance followed by Fisher’s protected least significant difference test using the Kaleida Graph software package (Hulinks, Tokyo, Japan).

## Results

### Propolis ethanol extract increases number of CD11b^+^, Gr-1^+^ MDSCs in the visceral adipose tissue of obese and lean mice

**Fig. 1:**
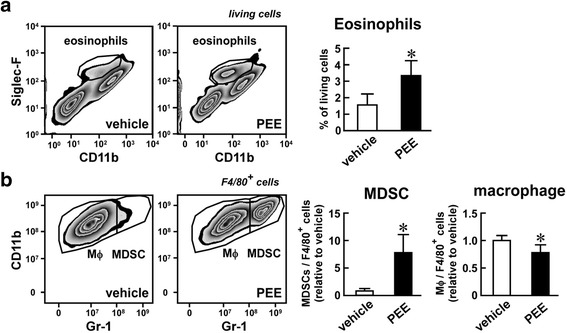
Effects of propolis ethanol extract (PEE) on the number of eosinophils, macrophages, and myeloid-derived suppressor cells (MDSCs) in the mesenteric adipose tissue of *ob/ob* mice. PEE (100 mg/kg, twice weekly) or vehicle was intraperitoneally injected into *ob/ob* mice for one month. Eosinophils, CD11b^+^, Gr-1^−^ macrophages, and CD11b^+^, Gr-1^+^ MDSCs were subsequently detected by FACS. **a** Eosinophils in the living cells. **b** Macrophages and MDSCs in F4/80^+^ myeloid cells. (Left scatter plots) Representative FACS images of four replicates. (Right graphs) Proportion of eosinophils in the living cells (**a**) and macrophages and MDSCs in F4/80^+^ myeloid cells (**b**). Data represents means ± SD of four samples. *P < 0.05 vs. vehicle-treated mice

Changes in the numbers of eosinophils and M1 macrophages were observed in a previous study four months after beginning injections of PEE (100 mg/kg i.p.) twice weekly [27]. In this study, we repeated the PEE dosage (100 mg/kg i.p., twice a week) and then increased it (250 mg/kg i.p., twice a week). The lower dose of PEE gave similar FACS data without any anorexic effects, but the higher dose repressed food intake and subsequently led to weight loss (data not shown). Therefore, we employed the 100 mg/kg dose of PEE going forward. Since our focus was on triggering cellular events that contribute to PEE-elicited anti-inflammation in adipose tissue, we collected the mesenteric adipose tissue of *ob/ob* mice after one month of treatment. One month of PEE treatment significantly reduced the blood glucose and total cholesterol levels of *ob/ob* mice, similar to approximately three months of treatment [27], although it did not affect body weight gain or blood triglyceride or non-esterified fatty acid levels (Additional files 1 and 2). FACS analysis indicated that PEE increased the number of eosinophils by about 2-fold in the mesenteric adipose tissue (Fig. 1a). While CD11b^+^, Gr-1^+^ MDSCs showed an increase in number (approx. 7.5-fold) in the mesenteric adipose tissue, the number of CD11b^+^, Gr-1^−^ macrophages were slightly reduced (approx. 0.8-fold; Fig. 1b). Relatively milder changes were observed in the MDSCs and macrophages in the epididymal adipose tissue (data not shown). Moreover, the preliminary experiment indicated that 50 mg/kg of PEE did not significantly raise the number of CD11b^+^, Gr-1^+^ MDSCs in the mesenteric and epididymal adipose tissue after one month of treatment. In summary, injections of 100 mg/kg PEE induced MDSCs in the visceral adipose tissues relatively early phase after PEE injection.

**Fig. 2:**
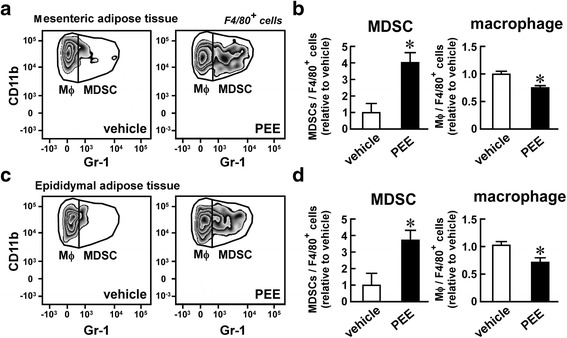
Effects of PEE on the number of macrophages and MDSCs in the visceral adipose tissue of high fat diet (HFD)-fed mice. HFD-fed C57BL/6 mice were intraperitoneally injected with PEE (100 mg/kg, twice weekly) or vehicle for one month. Subsequently, (**a**, **b**) mesenteric and (**c**, **d**) epididymal adipose tissue were subjected to FACS analysis of macrophages and MDSCs. **a**, **c** Representative FACS images of four replicates. Only F4/80^+^ living cells are shown. **b**, **d** Proportion of macrophages and MDSCs in F4/80^+^ myeloid cells from (**b**) mesenteric and (**d**) epididymal adipose tissue. Data represents means ± SD of four samples. *P < 0.05 vs. vehicle-treated mice

To examine whether the PEE-elicited induction of MDSCs was also observed in a feeding-induced obesity model, we treated HFD-fed obese mice with PEE. Low levels of CD11b^+^, Gr-1^+^ MDSCs were detected in the mesenteric and epididymal adipose tissues of vehicle-treated mice (Fig. 2). In contrast, MDSCs increased 4-fold in adipose tissue after PEE treatment for one month. PEE significantly lowered the number of CD11b^+^, Gr-1^−^ macrophages in HFD-fed obese mice (Fig. 2b and d), similar to the findings in *ob/ob* mice. In parallel with changes in the immune cell population in the adipose tissue, PEE administration significantly reduced circulating blood glucose levels with limited body weight gain changes (Additional files 1 and 2).

**Fig. 3:**
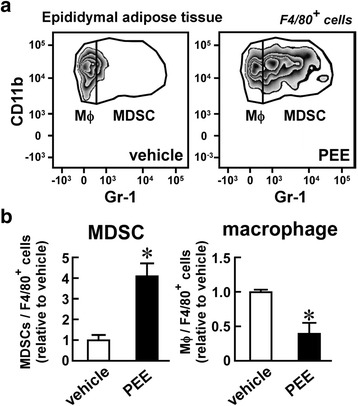
Effects of PEE on the number of macrophages and MDSCs in the epididymal adipose tissue of lean mice. Normal chow-fed C57BL/6 mice were intraperitoneally injected with PEE (100 mg/kg, twice weekly) or vehicle for one month. **a** Representative FACS images of four replicates. Only F4/80^+^ living cells are shown. **b** Proportion of macrophages and MDSCs in F4/80^+^ myeloid cells. Data represents means ± SD of four samples. *P < 0.05 vs. vehicle-treated mice

To assess whether PEE evokes MDSC accumulation in the adipose tissues in both obese and lean mice, we injected PEE or the vehicle on its own into lean C57BL/6 mice for one month and monitored CD11b^+^, Gr-1^+^ MDSCs in the adipose tissue. Mesenteric adipose tissue did not develop in lean mice, so we analyzed the epididymal adipose tissue. PEE did not affect the body weight gain and blood glucose levels of the lean C57BL/6 mice (Additional files 1 and 2). Conversely, the lean mice displayed a dramatic increase in CD11b^+^, Gr-1^+^ MDSCs in the epididymal adipose tissue after PEE administration, while the vehicle treatment failed to induce an increase (Fig. 3). In contrast, the CD11b^+^, Gr-1^−^ macrophages declined proportionately after PEE treatment (Fig. 3b). In summary, PEE has the potential to induce MDSCs in visceral fat regardless of the amount of body fat.

**Fig. 4:**
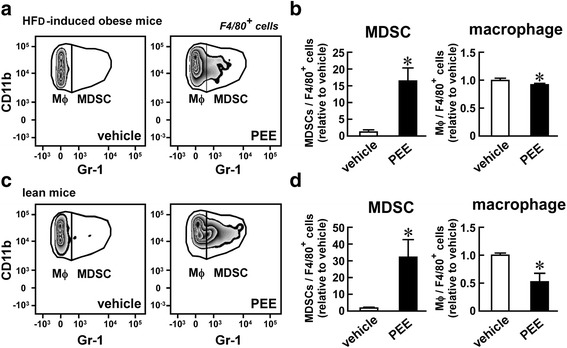
Effects of PEE on the number of macrophages and MDSCs in the peritoneum of HFD- and normal chow-fed mice. C57BL/6 mice were fed with (**a**, **b**) HFD or (**c**, **d**) normal chow. PEE (100 mg/kg, twice weekly) or vehicle was intraperitoneally injected into the mice for one month. Cells in the peritoneal cavity were harvested and subjected to FACS analysis for macrophages and MDSCs. **a**, **c** Representative FACS images of four replicates. Only F4/80^+^ living cells are shown. **b**, **d** Proportion of macrophages and MDSCs in F4/80^+^ myeloid cells in the peritoneum of (**b**) HFD-fed and (**d**) normal chow-fed mice. Data represents means ± SD of four samples. *P < 0.05 vs. vehicle-treated mice

To evaluate if the MDSC-inducing effect of PEE was limited to visceral adipose tissue, we also monitored the proportion of MDSCs in the peritoneal fluid. Figure 4a and b show the staining pattern of Gr-1 and CD11b from peritoneal cells of HFD-fed obese mice after one month’s treatment with PEE or the vehicle. As previously reported [33], most CD11b^+^ cells in the peritoneal fluid were Gr-1^−^ macrophages in the vehicle-treated mice. In contrast, PEE treatment resulted in a greater than 16-fold increase in CD11b^+^, Gr-1^+^ MDSCs in the peritoneal fluid of HFD-fed mice. A similar increase was also observed in lean mice after PEE treatment (Fig. 4c and d). Conversely, CD11b^+^, Gr-1^−^ macrophages decreased in the peritoneal fluid of both obese and lean mice after PEE treatment (Fig. 4b and d). In summary, these results suggest that MDSCs accumulate in both visceral adipose tissues and in the peritoneal cavity.

### Propolis ethanol extract directly induces CD11b^+^, Gr-1^+^ MDSCs from M1 macrophages

**Fig. 5:**
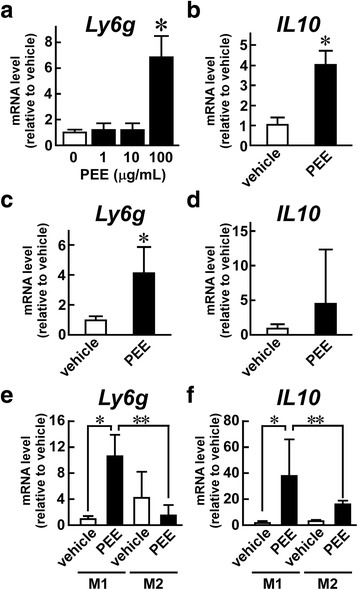
PEE induces MDSC markers in cultured macrophages. **a**, **b** Thioglycolate-elicited peritoneal macrophages, (**c**, **d**) J774.1 cells, and (E, F) GM-CSF-elicited M1 and M-CSF-elicited M2 macrophages were stimulated with PEE (**a**; 1, 10, or 100 μg/mL: **b**-**f**; 100 μg/mL) for 24 h. Subsequent qRT-PCR for (**a, c, e**) *Ly6g* and (**b**, **d**, **f**) *Il10* was performed. Values were normalized to (**a-d**) *Hprt1* mRNA or (**e**, **f**) 18 s rRNA levels, and represented as the means ± SD of (**a**, **b**, **e**, **f**) 4 or (**c**, **d**) 3 samples. *P < 0.05 vs. vehicle-treated mice; **P < 0.05 vs. M2 macrophages

Although the *in vivo* experimental data showed the efficacy of PEE on MDSC induction, the direct cellular target of PEE remained unclear. We hypothesized that MDSCs might be derived from macrophages after PEE treatment because the number of CD11b^+^, Gr-1^−^ macrophages decreased in parallel with increasing CD11b^+^ Gr-1^+^ MDSC numbers in the visceral adipose tissue and peritoneum of PEE-treated mice. To test this hypothesis, we treated peritoneal macrophages with PEE (1, 10, 100 μg/mL) or with the vehicle, and then monitored the transcripts for levels of Gr-1 (official gene symbol, *Ly6g*) and IL-10 (official gene symbol, *Il10*). The 1 μg/mL and 10 μg/mL doses of PEE did not raise the levels of *Ly6g* mRNA (Fig. 5a), similar to the vehicle-treated cells. However, the 100 μg/mL dose significantly upregulated the expression of *Ly6g*. The same dose of PEE also increased the expression of the immunosuppressive cytokine *Il10* (Fig. 5b), which is abundantly expressed in MDSCs [10]. Similar increases of *Ly6g* and *Il10* were also observed in the macrophage-like cell line J774.1 after treatment with PEE (Fig. 5c and d).

Macrophages are roughly classified into M1 and M2 macrophages. We investigated which type of macrophage was the dominant source of PEE-elicited MDSCs by preparing M1 and M2 macrophages from bone marrow cells after treatment with GM-CSF and M-CSF, respectively. Treatment with PEE elevated *Ly6g* expression in M1 macrophages but not in M2 macrophages (Fig. 5e). Similarly, PEE increased *Il10* expression in M1 macrophages more than in M2 macrophages, although the basal expression level of this cytokine tended to be higher in M2 macrophages than in M1 macrophages (Fig. 5f). Since peritoneal macrophages and J774.1 cells exhibit the cellular properties of M1 macrophages, PEE has the potential to induce MDSCs from M1 macrophages.

### Kaempferol induces MDSCs from macrophages

**Fig. 6:**
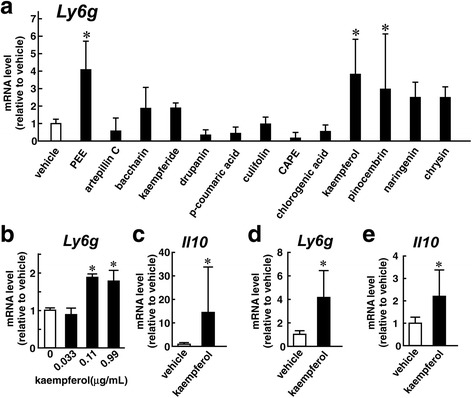
Effects of resinous compounds on the expression of MDSC markers in cultured macrophages. **a**-**c** Thioglycolate-elicited macrophages or (**d**, **e**) J774.1 cells were treated with vehicle, PEE (100 μg/mL), or twelve resinous compounds involved in PEE for 24 h and subsequently subjected to qRT-PCR analysis. **a** Comparisons of the effects of the compounds on *Ly6g* expression. Doses of each compound are described in the Results. **b**-**e** Effects of kaempferol (**b**, 0.03, 0.1, or 1 μg/mL; **c**-**e**, 0.1 μg/mL) on (**b**, **d**) *Ly6g* and (**c**, **e**) *Il10* expression in (**b**, **c**) peritoneal macrophages and (**d**, **e**) J774.1 cells. Values were normalized to *Hprt1* mRNA levels, and represented as means ± SD of (**a**) 4, (**b**) 3–4, (**c**) 3–5, or (**d**, **e**) 7 samples

We examined which resinous compounds could induce MDSCs from macrophages. We treated thyoglycolate-elicited peritoneal macrophages with twelve compounds whose dose was determined based on their proportional ratio in PEE. As shown in Fig. 6a; kaempferol (0.11 μg/mL) and pinocembrin (0.037 μg/mL) increased *Ly6g* expression in the cells, while artepillin C (9.5 μg/mL), drupanin (1.4 μg/mL), CAPE (0.14 μg/mL), baccharin (3.5 μg/mL), kaempferide (1.7 μg/mL), culifolin (0.23 μg/mL), chlorogenic acid (0.12 μg/mL), p-coumaric acid (1.2 μg/mL), naringenin (0.019 μg/mL), and chrysin (2.9 ng/mL) did not cause any changes in *Ly6g* mRNA levels. We further analyzed the effects of kaempferol on macrophages because kaempferol showed the largest influence on *Ly6g* expression. Figure 6b shows the dose-dependent effects of kaempferol on *Ly6g* expression in peritoneal macrophages. Although 0.03 μg/mL of kaempferol did not modulate *Ly6g* expression, 0.1 μg/mL and 1 μg/mL of the compound significantly induced *Ly6g* in the macrophages. Moreover, *Il10* mRNA also accumulated in the cells after treatment with 0.1 μg/mL of kaempferol (Fig. 6c). Similarly, kaempferol increased expression of *Ly6g* and *Il10* in J774.1 cells (Fig. 6d and e). Therefore, it is likely that kaempferol is involved in the PEE-elicited MDSC induction.

### Kaempferol causes accumulation of MDSCs in the mesenteric adipose tissue of mice

**Fig. 7:**
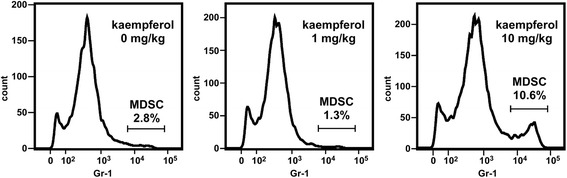
Effects of kaempferol on the number of MDSCs in epididymal adipose tissue of mice. Kaempferol (1 or 10 mg/kg, twice weekly) or vehicle was intraperitoneally injected to lean C57BL/6 mice for two months. The vascular stromal fraction was stained, and subsequently subjected to FACS analysis. Representative FACS images of CD11b^+^ cells in triplicates are shown. Proportion of CD11b^+^, Gr-1^+^ MDSCs in all CD11b^+^ cells and doses of kaempferol are also described

We evaluated the effects of kaempferol on the myeloid population in the adipose tissue of mice. In preliminary experiments, C57BL/6 mice were given intraperitoneal injections of the vehicle, 1 mg/kg or 10 mg/kg kaempferol twice weekly for one month, with no significant induction of MDSCs in the epididymal adipose tissue. However, two months of treatment with 10 mg/kg of kaempferol significantly increased the MDSCs in the epididymal adipose tissue of the C57BL/6 mice, although treatment for the same period with 1 mg/kg did not affect the number of MDSCs (Fig. 7). After two months of treatment, neither 1 mg/kg nor 10 mg/kg of kaempferol had significant effects on body weight gain or blood glucose levels in lean C57BL/6 mice (Additional files 1 and 2). Nevertheless, it is likely that kaempferol is one of the agents inducing MDSCs in visceral adipose tissue.

## Discussion

PEE represses the progression of type 2 diabetes in mouse and rat models [27, 34–37]. This beneficial effect of PEE on type 2 diabetes has been observed in several ways. For example, PEE suppresses feeding through the secretion of leptin from adipocytes [37]. PEE also stimulates glucose uptake in skeletal muscle by recruiting glucose transporter 4 to the plasma membrane [38]. Previously, we demonstrated that intraperitoneal injection of PEE attenuated adipose tissue inflammation in leptin-deficient *ob/ob* mice resulted in a dramatic repression of metabolic deterioration [27]. In this study, we demonstrate that PEE causes a rapid accumulation of MDSCs in the visceral adipose tissue of mice. MDSCs have the potential to repress inflammation in adipose tissues [12], therefore it is likely that PEE alleviates inflammation through the induction of MDSCs. MDSCs accumulate in the adipose tissue of obese and lean mice, implying that PEE has the potential to prevent both the onset and progression of type 2 diabetes.

In this study, MDSCs accumulated in visceral adipose tissue and in peritoneal fluid. The source of the PEE-elicited MDSCs is still unclear; they are possibly recruited from other tissue such as bone marrow or differentiated from resident macrophages or circulating monocytes. We demonstrated that PEE directly induces MDSCs from cultured peritoneal macrophages. Peritoneal macrophages can be divided into two populations: F4/80^high^ MHC-II^−^ predominant in intact mice, and F4/80^mid^ MHC-II^+^ predominant in thyoglycolate-elicited mice [39, 40]. Since *in vivo* F4/80^high^ MHC-II^−^ and *in vitro* F4/80^mid^ MHC-II^+^ macrophages can produce nitric oxide in response to LPS stimulation, both cell types display characteristics of M1 macrophages [39]. On the other hand, PEE also increases the expression of the MDSC markers *Ly6g* and *Il10* in the cloned cell line J774.1, which constantly express the M1 macrophage markers tumor necrosis factor (TNF) -α and inducible nitric oxide synthase [41]. PEE induced MDSC markers in GM-CSF-treated bone marrow cells (M1 macrophages), but not in M-CSF-treated cells (M2 macrophages). These results collectively suggest that the PEE-elicited MDSCs originate from M1 macrophages in the visceral organs or blood.

Of the twelve resinous compounds of PEE that were assessed, kaempferol increased *Ly6g* and *Il10* mRNA in cultured macrophages when used at a dosage comparable to that found in PEE. Moreover, repeated injections of kaempferol increased the number of CD11b^+^, Gr-1^+^ MDSCs in the adipose tissue of mice. These results suggest that kaempferol is the compound participating in the PEE-elicited MDSC induction. Kaempferol has been shown to display anti-inflammatory effects mediated by PPARγ activation [42], while PPARγ has also been shown to participate in the induction of MDSCs in the peritoneum after marijuana cannabidiol treatment [43]. A previous paper suggested that PPARγ is involved in the restoration of metabolic disorder by propolis or kaempferol treatment [44–46]. Therefore, PPARγ may also be critical for MDSC induction in visceral adipose tissue after PEE or kaempferol treatment.

In addition to *Ly6g* and *Il10*, MDSCs are known to express arginase-1 (*Arg1*), which attenuates nitric oxide synthesis [47]. In our preliminary experiments, PEE increased the expression of *Arg1* in J774.1 cells, as well as in bone marrow-derived M1 macrophages. Conversely, kaempferol failed to elevate *Arg1* expression in J774.1 cells, although it did increase *Arg1* mRNA in isolated macrophages. The differing effects of PEE and kaempferol on *Arg1* mRNA might be attributed to other chemical compounds present in PEE. In support of this idea, naringenin was shown to potentiate *Arg1* expression in macrophages [48]. Further studies are required to assess the combinatory effects of the chemical compounds in PEE on the expression of each molecule predominantly expressed in MDSCs, as well as on mice with different metabolic states.

In *in vitro* culture systems, a combined treatment with IL-6 and GM-CSF induced MDSCs from bone marrow myeloid progenitor cells [49]. Our results indicate that MDSCs can also be obtained from differentiated M1 macrophages *in vitro*. M1 macrophages are major participants in the pathogenesis of adipose tissue inflammation through the secretion of proinflammatory molecules, such as TNF-α and IL-6 [50, 51]. Thus, the PEE- and kaempferol-elicited M1 macrophage reduction potentially contributes to the repression of adipose tissue inflammation. Together with the strong immune-suppressive phenotype of MDSCs [9], PEE and kaempferol provide two additional advantages: the decrement of proinflammatory cells and the increment of anti-inflammatory cells.

Although the intraperitoneal injection of PEE strongly induced MDSCs in the visceral tissues of mice, there are several preventive and therapeutic issues to address regarding type 2 diabetes in humans. There are some differences in the detailed characteristics of MDSCs between human and mice, such as surface markers [9], and it is recommended that further studies evaluate if PEE induces MDSCs in humans. It is also recommended that further studies examine whether oral administration, rather than intraperitoneal injection, causes accumulation of MDSCs in adipose tissue, in consideration of the safety and accessibility of medication.

In this research, we conducted animal experiments to evaluate the *in vivo* effects of PEE. The original aim of this study was to identify the immunosuppressive cellular population in adipose tissue, and *in vitro* models could not replace mice. All efforts were made to reduce the number of animals used compared to previous studies [27, 37] and we concluded that four animals per experiment appears to be sufficient to examine the effects of PEE on adipose tissue inflammation in C57BL/6 mice.

## Conclusion

PEE and its constituent kaempferol directly induce MDSCs from M1 macrophages *in vitro* and *in vivo*. MDSCs rapidly appeared in visceral adipose tissue after PEE and kaempferol treatment, indicating the possible involvement of MDSCs in PEE- or kaempferol-elicited anti-diabetic effects. Further studies are needed to clarify the cellular and molecular networks that are activated by accumulated MDSCs in the adipose tissue. Considering that propolis has effects on the central nervous system as well as on major energy-consuming tissues such as the liver and skeletal muscle [34, 36–38], our data supports the idea that propolis is a multi-target agent in preventing metabolic disorders.

## Additional files

Additional file 1: Mean body weight of animals for this study before and after treatments. (XLSX 18 kb)

Additional file 2: Blood test data of animals for this study after treatments. (XLSX 12 kb)

Additional file 3: Raw data of FACS and qRT-PCR analyses. Statistical analysis data are also included. (XLSX 5920 kb)

Additional file 4: Gating processes of FACS analysis. (PDF 516 kb)

## Abbreviations

Arg1: Arginase-1
CAPE: Caffeic acid phenethyl ester
DMEM: Dulbecco’s modified Eagle medium
DMSO: Dimethyl sulfoxide
FACS: Fluorescence activated cell sorting
FCS: Fetal calf serum
GM-CSF: Granulocyte macrophage colony-stimulating factor
HFD: High fat diet
Hprt1: Hypoxanthine-guanine phosphoribosyltransferase 1
IL: Interleukin
M-CSF: Macrophage colony- stimulating factor
MDSC: Myeloid-derived suppressor cell
PEE: Propolis ethanol extract
qRT-PCR: Quantitative reverse transcription-polymerase chain reaction
TGF: Transforming growth factor
TNF: Tumor necrosis factor
VSF: Vascular stromal fraction
