# Supplementary material for: Brazilian propolis ethanol extract and its component kaempferol induce myeloid-derived suppressor cells from macrophages of mice in vivo and in vitro
Source: BMC Complement Altern Med. 2018 May 2;18:138. doi: 10.1186/s12906-018-2198-5 (PMC5930496; doi:10.1186/s12906-018-2198-5)
Supplement: Supplementary file 4 — Gating processes of FACS analysis. (PDF 516 kb) [file 12906_2018_2198_MOESM4_ESM.pdf]

Mesenteric adipose tissue of C57BL/6 *ob/ob* mice (eosinophil)

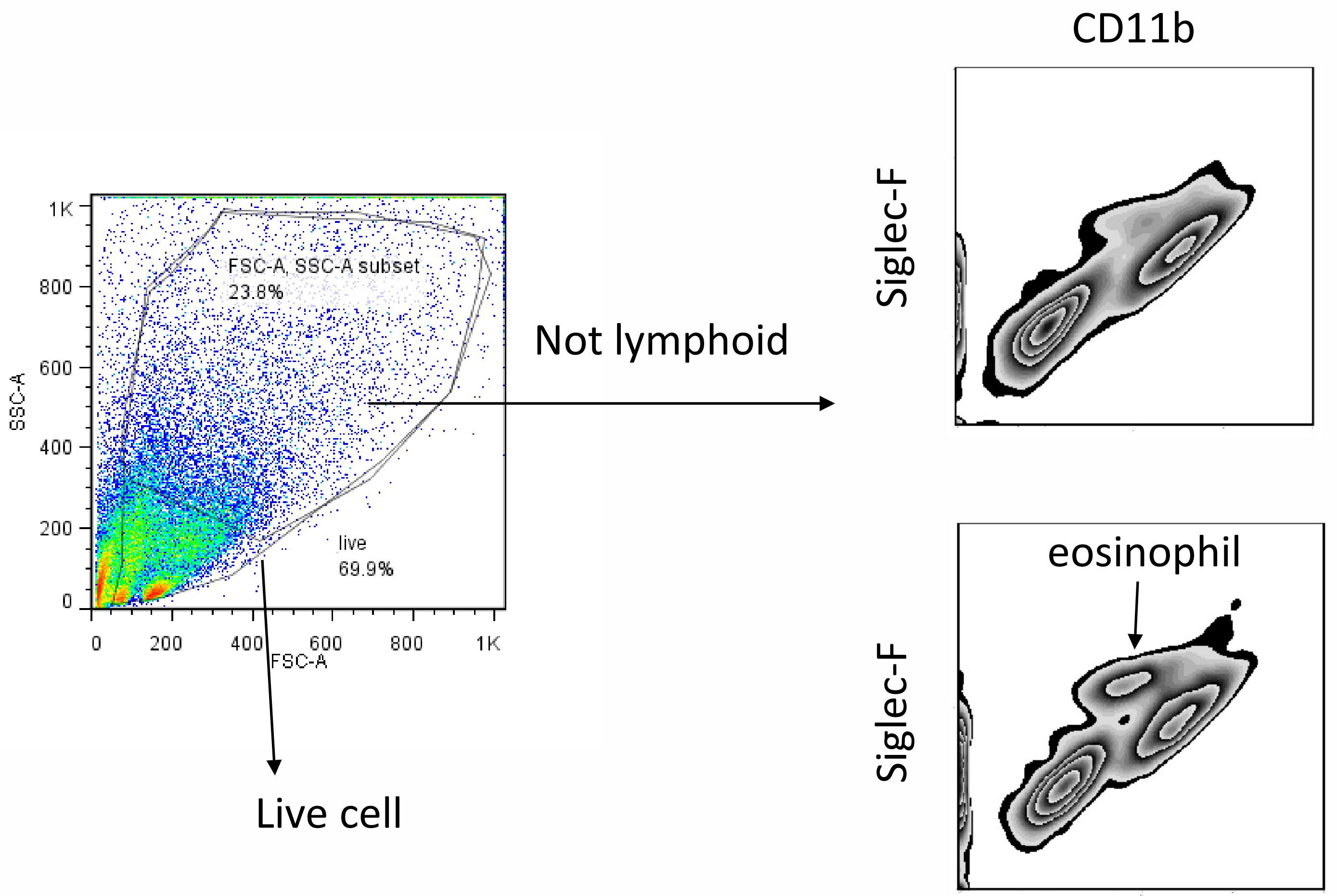

Figure 1A left
(vehicle)

Figure 1A right
(PEE)

Data were obtained using a FACS Canto II (BD Bioscience).

Mesenteric adipose tissue of C57BL/6 *ob/ob* mice (macrophage and MDSC)

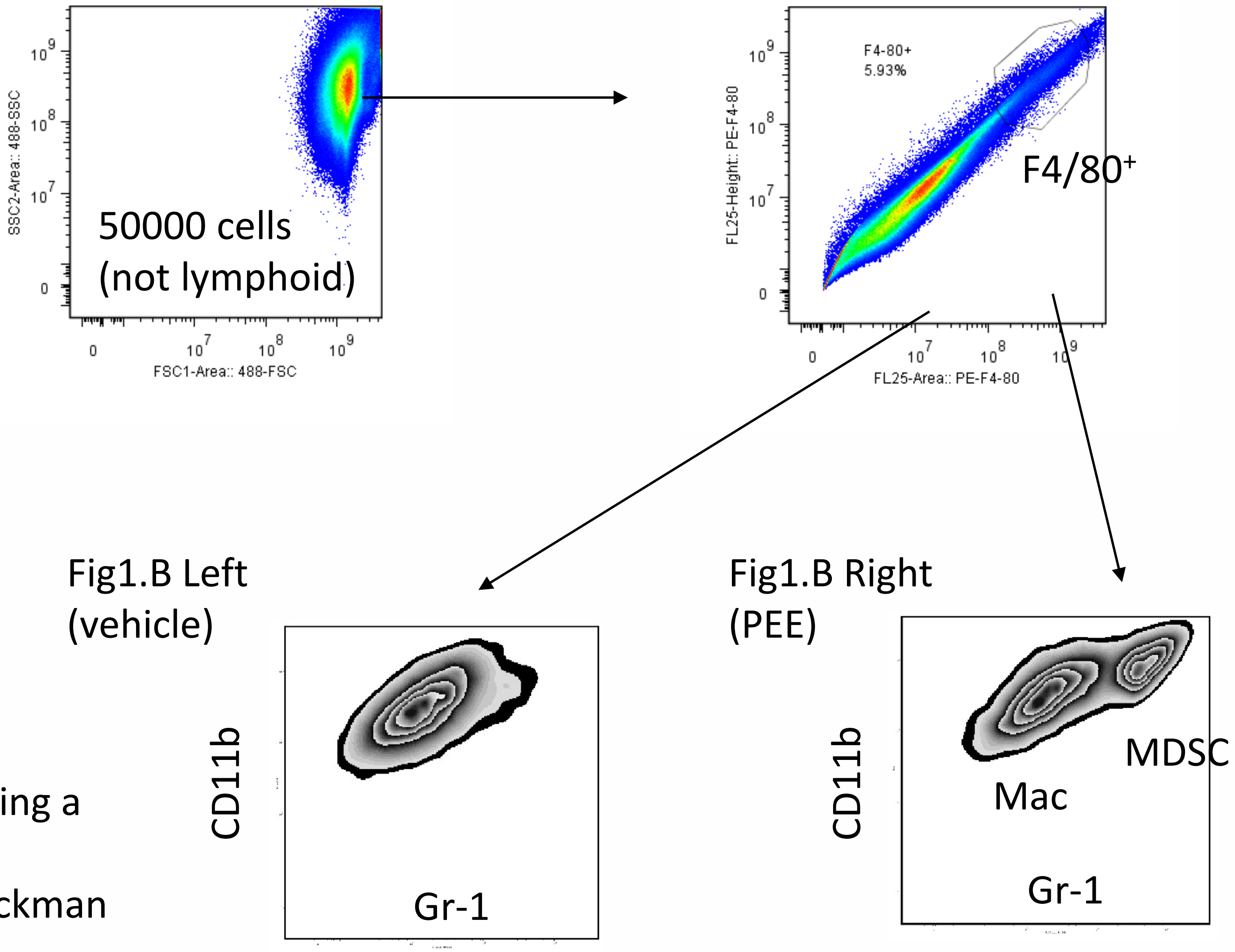

Data were obtained using a MoFlo Astorios(Beckman Coulter).

Mesenteric adipose tissue of HFD-induced obese C57BL/6 mice

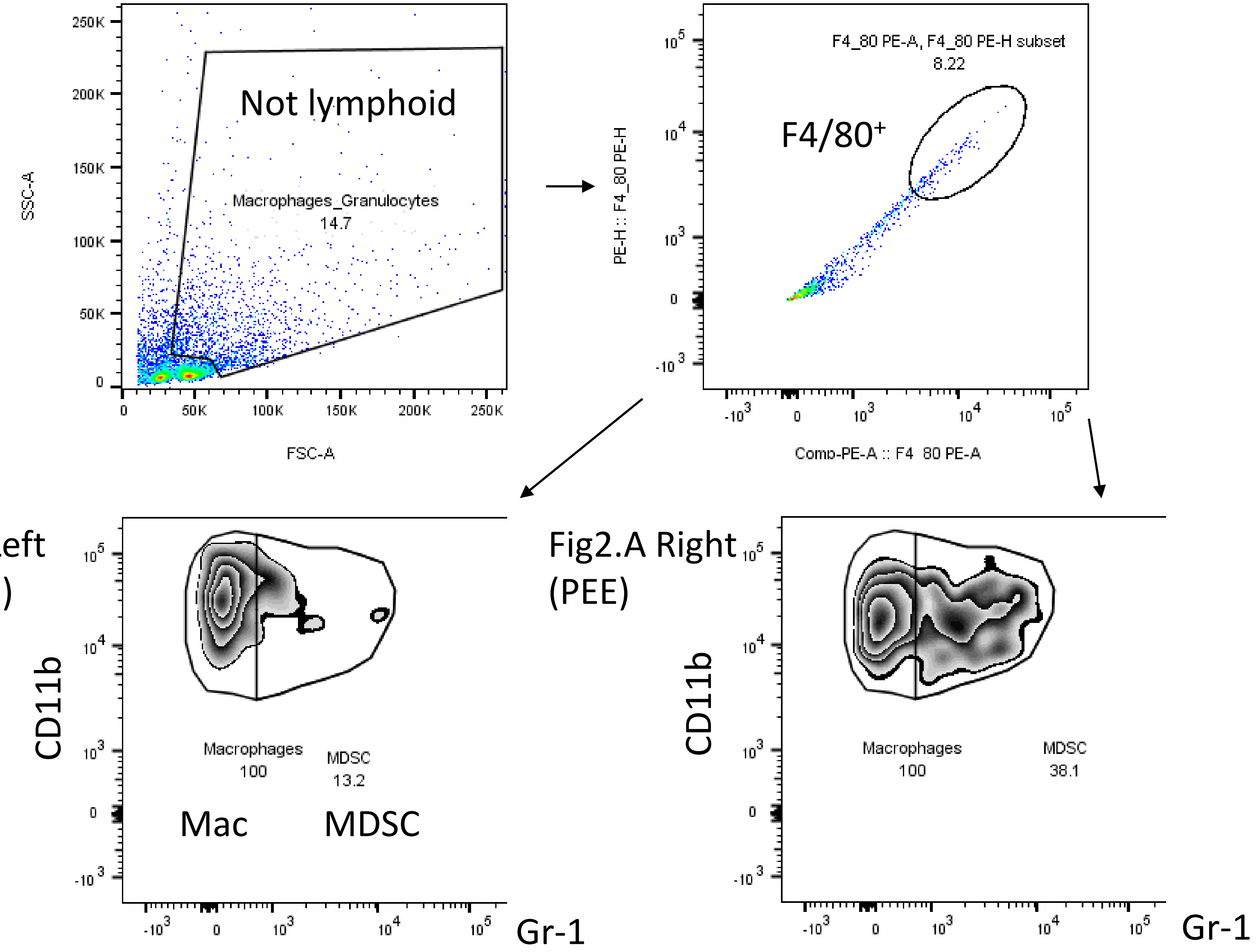

Data were obtained using FACS Canto II (BD Bioscience).

Epididymal adipose tissue of HFD-induced obese C57BL/6 mice

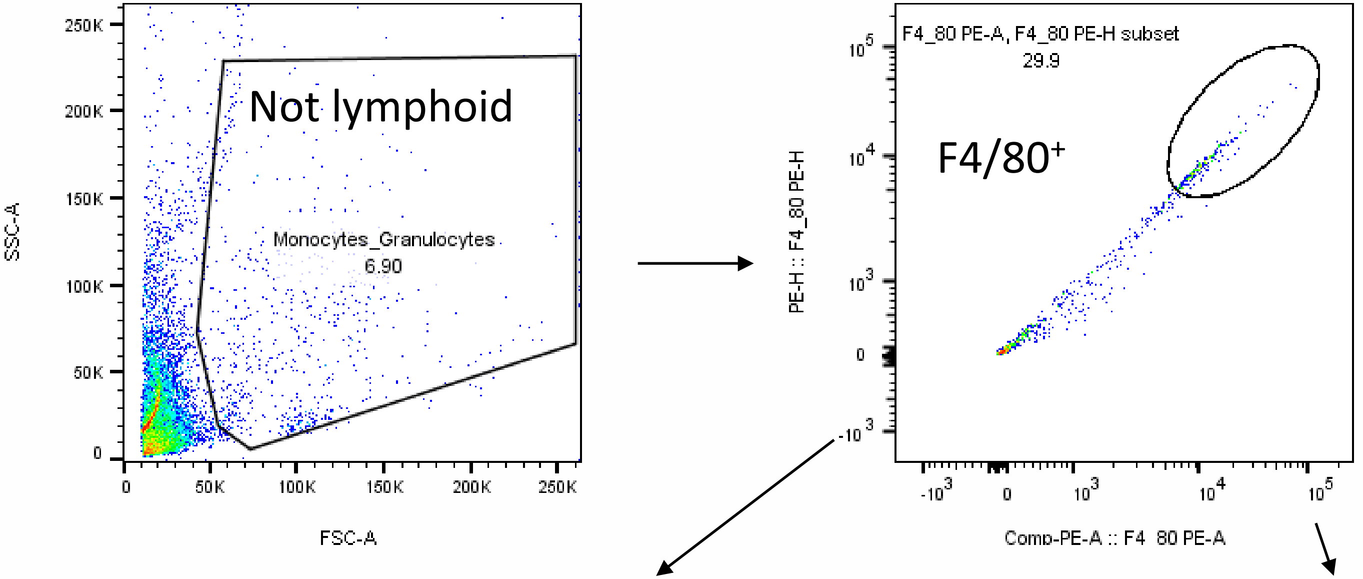

Fig2.C Left
(vehicle)

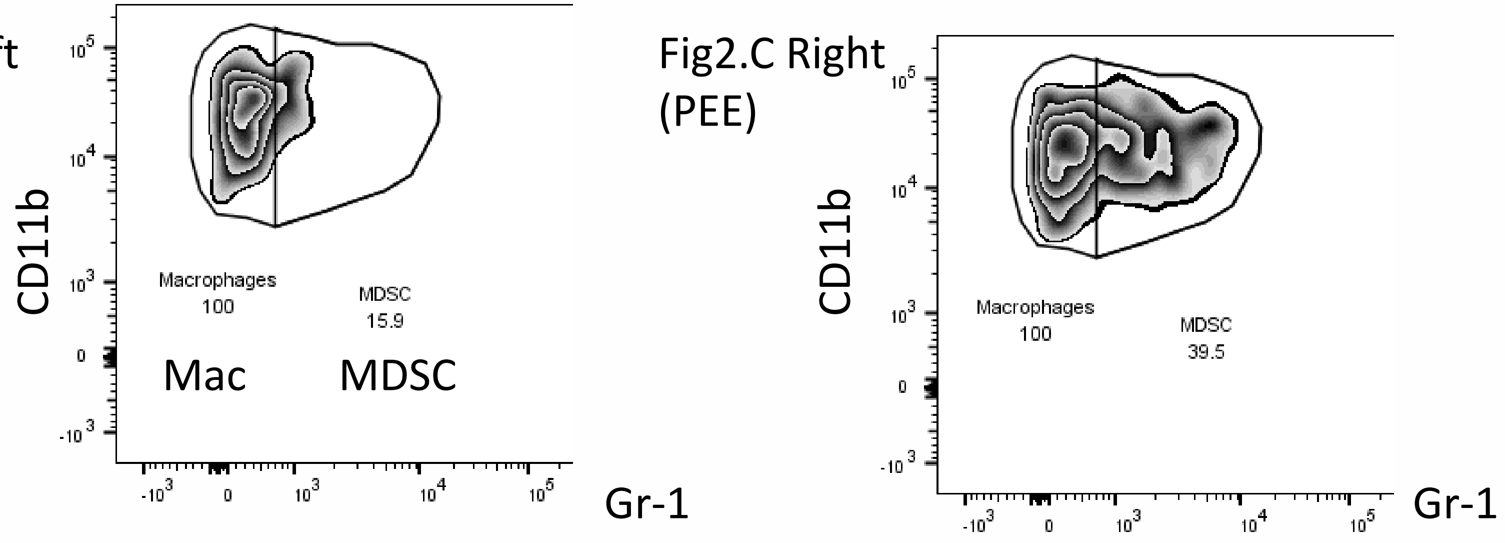

Data were obtained using FACS Canto II (BD Bioscience).

Epididymal adipose tissue of lean C57BL/6 mice

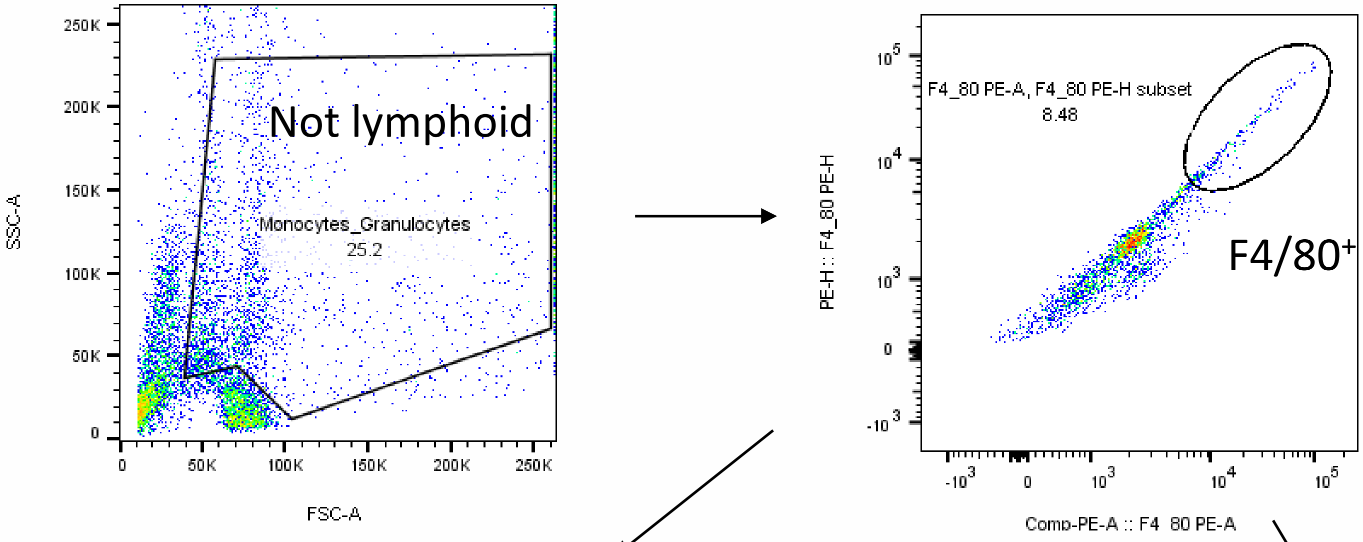

Fig3.A Left
(vehicle)

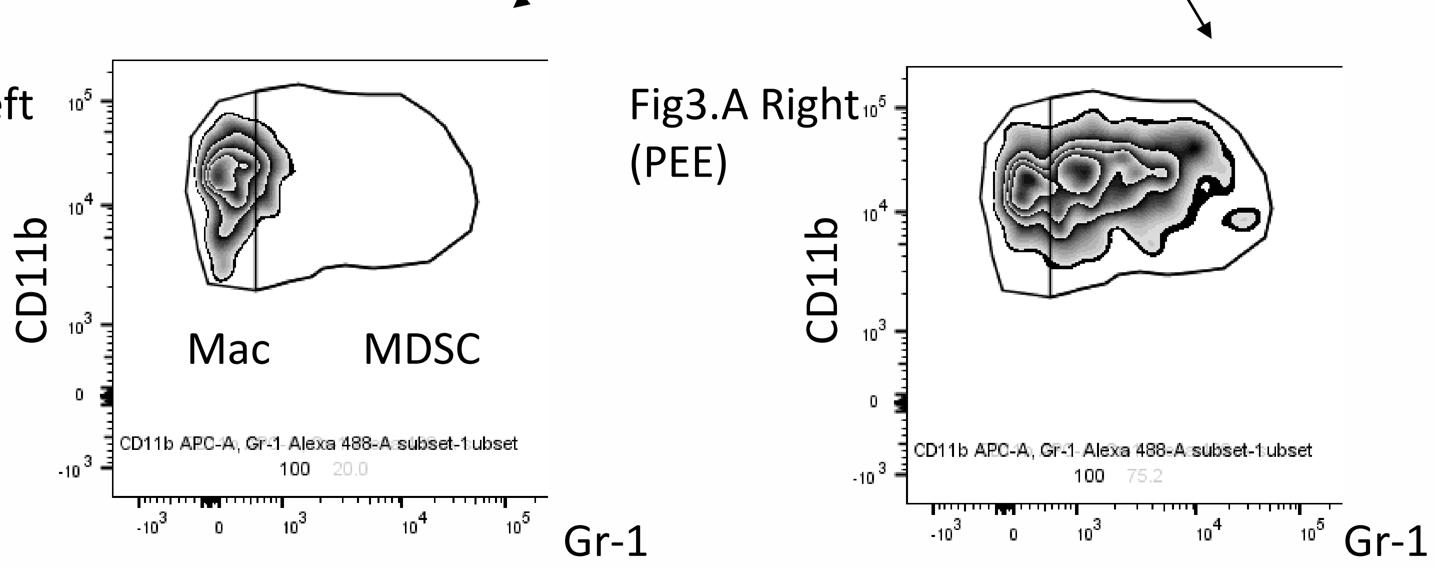

Data were obtained using a FACSVerse(BD Bioscience).

Peritoneal cells of HFD-induced obese C57BL/6 mice

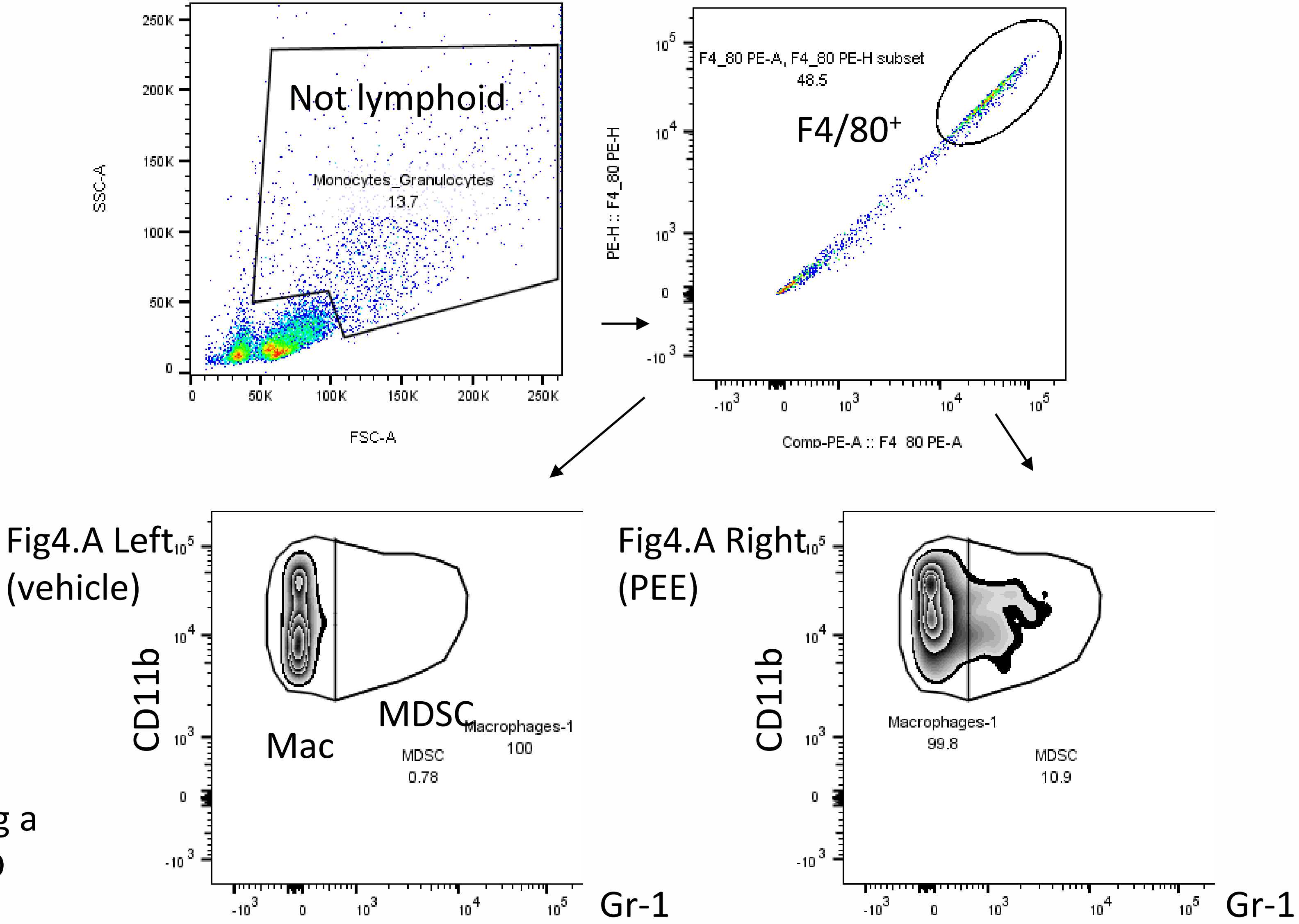

Data were obtained using a FACSVerse (BD Bioscience).

Peritoneal cells of lean C57B/6 mice

Fig4.C Left
(vehicle)

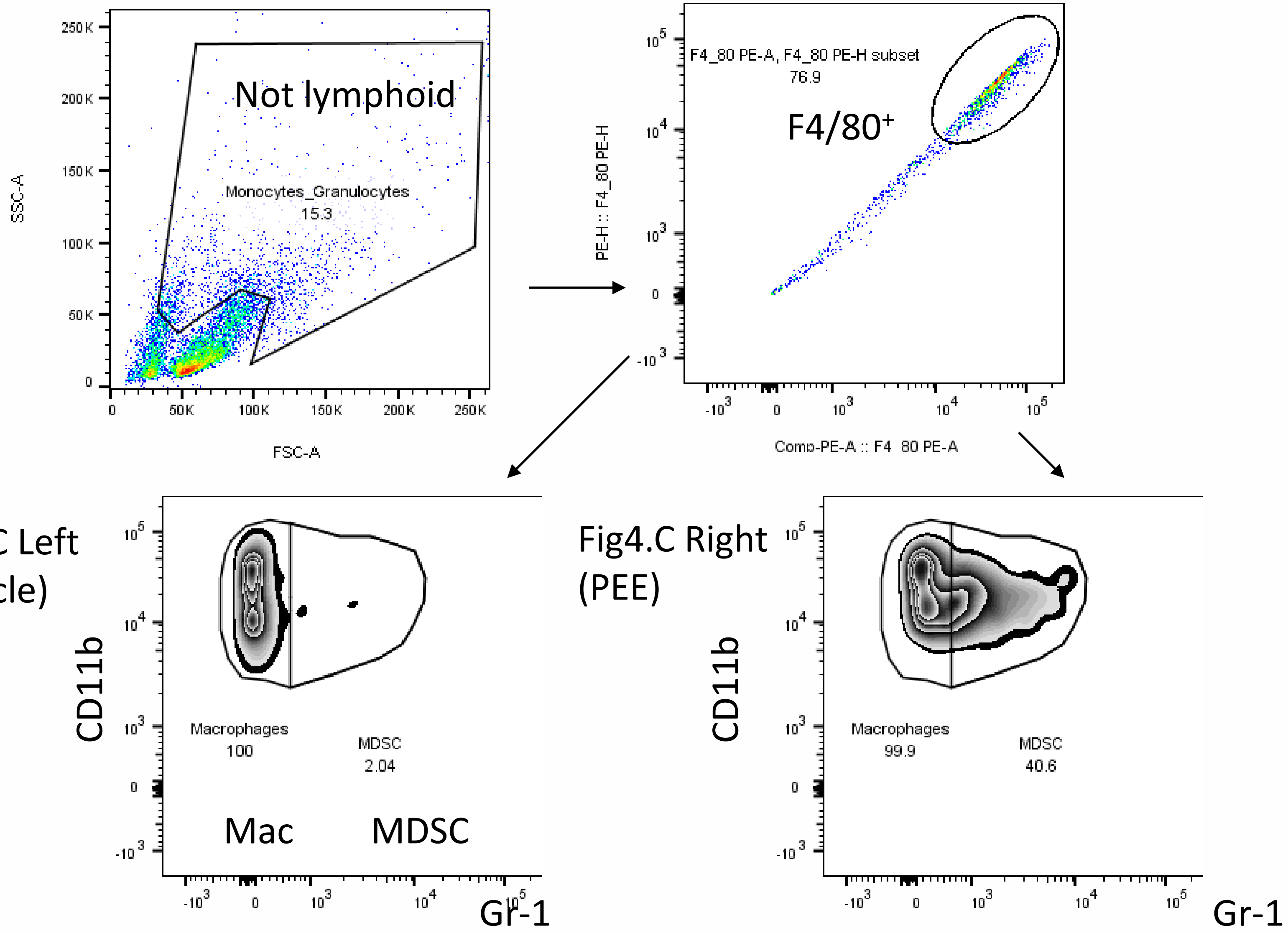

Data were obtained using a FACSVerse(BD Bioscience).

Epididymal adipose tissue of lean C57BL/6 mice (kaemferol injection)

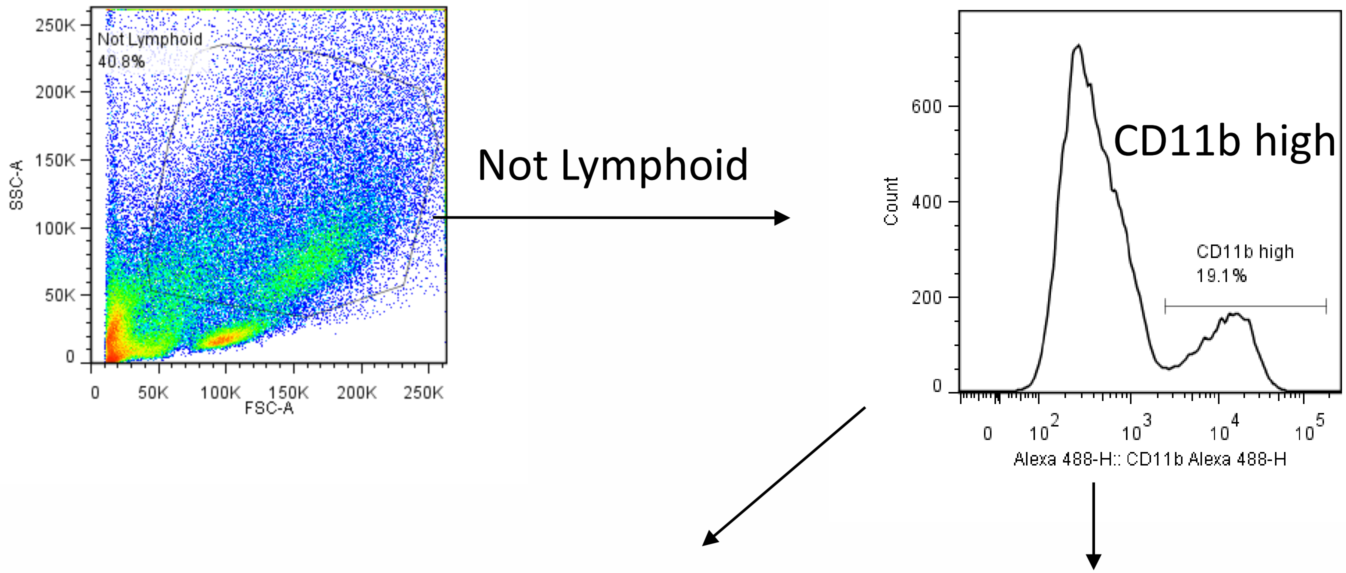

Figure 7

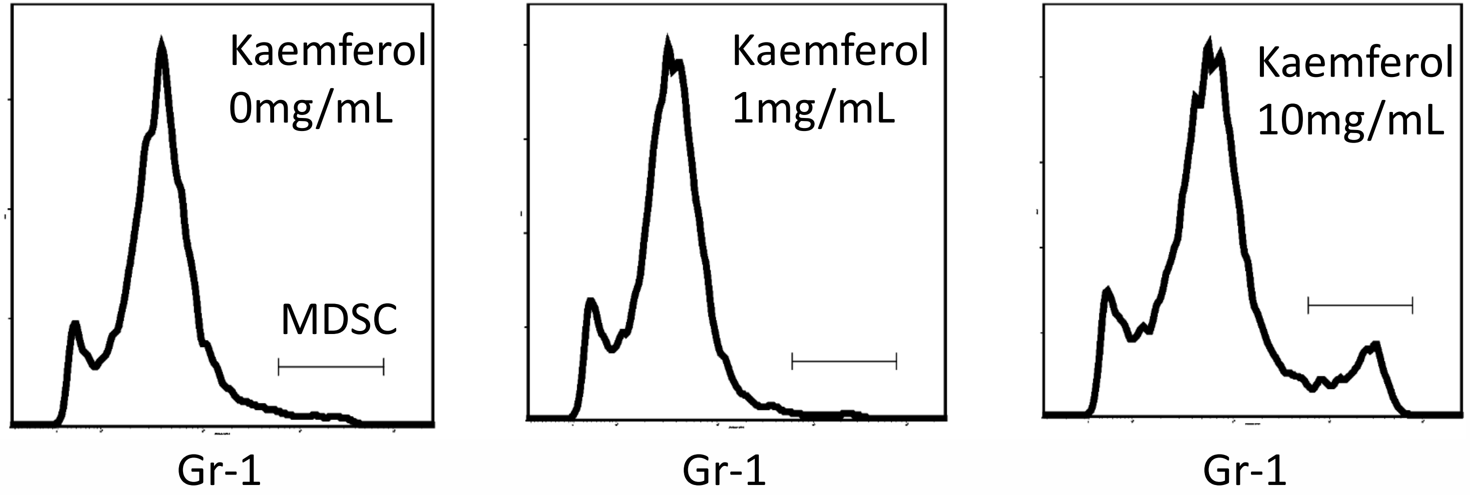

Data were obtained using a FACSVerse(BD Bioscience).
